# Immune Infiltration Landscape in Osteosarcoma: Clinical Implications for Prognosis and Therapy

**DOI:** 10.1002/cnr2.70495

**Published:** 2026-02-12

**Authors:** Yuerong Wang, Xueni Liu, Guojin Xie, Zheqian Li

**Affiliations:** ^1^ Department of Clinical Laboratory Children's Hospital of Nanjing Medical University Nanjing China

**Keywords:** immune cell infiltration subtypes, immunotherapy, osteosarcoma, prognostic biomarkers, tumor microenvironment

## Abstract

**Background:**

With survival rates for osteosarcoma largely unchanged for 40 years—especially in metastatic/recurrent cases. While the immune microenvironment is believed to play a crucial role, the heterogeneity of immune cell infiltration (ICI) and its precise impact on prognosis and therapeutic response remain poorly characterized. There is a lack of a robust, ICI‐based scoring system to stratify patients and identify novel therapeutic targets. This study aimed to identify the characteristics of ICI subtypes for evaluating prognosis and therapeutic benefits.

**Methods:**

Based on gene expression and clinical data from TARGET and GEO databases, ICI was characterized using ESTIMATE and CIBERSORT, leading to the development of a prognostic ICI score via PCA that stratified patients into high‐ and low‐score groups. Key genes associated with this score were subsequently identified through WGCNA and further validated by constructing a prognostic prediction model. Finally, the ICI score demonstrated significant predictive value for metastasis and HUVOS grade across clinical cohorts.

**Results:**

We identified three ICI subtypes with distinct prognostic values. Patients with higher ICI scores showed improved survival and were enriched in CD8^+^ T cells, monocytes, M1 macrophages, M2 macrophages, activated dendritic cells, resting mast cells, and activated mast cells. Three key genes—WAS, ARHGAP30, and PARVG—were associated with metastasis, Huvos grade, and macrophage‐specific expression and served as potential prognostic biomarkers.

**Conclusion:**

This study highlights the prognostic and therapeutic relevance of immune infiltration in osteosarcoma. The ICI score and key genes offer insights into tumor heterogeneity and potential therapeutic targets, particularly in modulating macrophage polarization and enhancing antitumor immunity. Limitations include retrospective data and lack of functional validation. Future work should focus on experimental verification and clinical translation of these immune biomarkers.

## 1 Background

Osteosarcoma (OS), the most common primary malignant bone tumor, originates from the abnormal proliferation of osteoid tissue and immature bone cells and predominantly occurs in children and adolescents, particularly in areas characterized by active bone growth [1]. Although the current standard treatment for osteosarcoma is a neoadjuvant chemotherapy‐surgery‐consolidation chemotherapy treatment mode, it has improved outcomes for localized disease; the prognosis for patients with metastatic or recurrent OS remains poor, with relapse rates of 30%–50% accounting for the majority of deaths [2, 3, 4, 5]. The highly aggressive and metastatic nature of OS underscores the urgent need for more effective therapeutic strategies.

The tumor microenvironment (TME) has gained recognition as a crucial contributor to OS progression and therapy resistance [6, 7, 8], fostering tumor growth, immunosuppression and enabling immune evasion [5, 9]. Recent efforts have focused on targeting the TME through immunotherapies such as checkpoint inhibitors, cytokine treatments, adoptive cell transfer, and cancer vaccines to enhance antitumor responses while minimizing toxicity [10, 11, 12]. Furthermore, numerous studies within the past 5 years have sought to elucidate OS tumorigenesis, block pro‐tumor signaling, counteract immune evasion, and overcome drug resistance [4]. Despite these advances, the immune infiltration landscape in OS remains poorly characterized, hindering prognostic accuracy and the effective application of immunotherapy [13, 14].

In this study, we aim to address this gap by performing a comprehensive analysis of the ICI patterns in osteosarcoma. Using transcriptomic data from multiple cohorts and computational approaches including CIBERSORT and ESTIMATE, we identify distinct ICI subtypes and correlate these with clinical outcomes. We further establish an ICI scoring system to stratify patients into high‐ and low‐risk groups, identify prognostic gene signatures through differential expression and WGCNA, and pinpoint key hub genes associated with immune response. Our findings provide new insights into the immunobiology of OS and support the development of personalized immunotherapeutic strategies.

## 2 Materials and Methods

**FIGURE 1:**
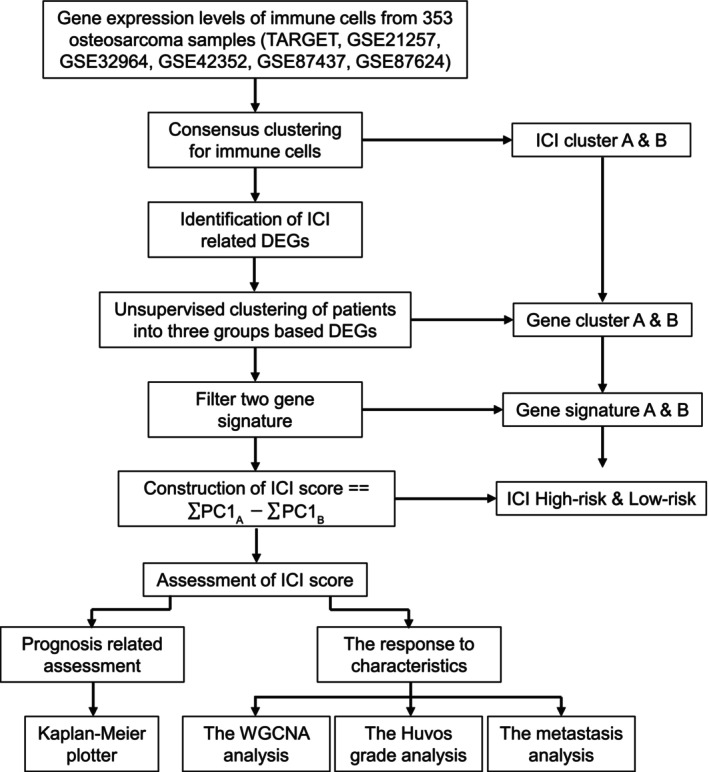
The flowchart of the whole study.

The flowchart of the whole study was presented in Figure 1. We acquired gene expression profiles of immune cells from the Therapeutically Applicable Research to Generate Effective Treatments (TARGET) and Gene Expression Omnibus (GEO) databases, and performed consensus clustering on these immune cell data. Leveraging the differentially expressed genes (DEGs) identified, we conducted unsupervised clustering to stratify patients into two distinct genomic clusters, designated as Cluster A and Cluster B. Among these genes, those positively associated with the gene cluster were designated as ICI gene signature A, while those negatively associated were designated as ICI gene signature B. Subsequently, principal component analysis (PCA) was performed to calculate the ICI gene signature score using the formula:

ICIscore=∑PC1A−∑PC1B.

A detailed description of the complete methodology is provided in the subsequent sections.

### 2.1 Osteosarcoma Cohorts and Processing

A total of 353 osteosarcoma samples were obtained from the TARGET (https://www.cancer.gov/ccg/research/genome‐sequencing/target), including RNA‐sequencing transcriptomic and clinical data. Microarray data (GSE21257, GSE32964, GSE42352, GSE87437, GSE87624) were downloaded from GEO (https://www.ncbi.nlm.nih.gov/geo). Patients without full survival information were excluded from our cohort. The count values from the TARGET dataset were transformed into transcripts per kilobase million (TPM) values, as previously described [15]. ComBat algorithm was utilized to reduce the batch effects caused by nonbiotech bias between different datasets [16].

### 2.2 Consensus Clustering for Immune Cells

Gene expression levels of immune cells in osteosarcoma were quantified using CIBERSORT R package [17], and ESTIMATE algorithm [18] was used to evaluate tumor purity, stromal, and immune score. Then, the hierarchical agglomerative clustering of osteosarcoma was performed in accordance with ICI pattern. We conducted the ConsensuClusterPlus package [19] to execute the steps above and repeated 1000 times to provide stabilized classification results.

### 2.3 DEGs Associated With ICI Phenotype

We stratified patients into three ICI clusters based on the infiltration levels of immune cells to identify differentially expressed genes (DEGs) associated with distinct ICI patterns. Using limma R, significant cutoff criteria were established as adjusted *p* < 0.05 [20]. Then, the ClusterProfiler R package was employed to perform functional annotation for every gene with a cutoff value of false discovery rate (FDR) < 0.05 [21].

### 2.4 Dimension Reduction and Construction of ICI Score

In our study, we performed unsupervised clustering to stratify patients into two genomic clusters (Clusters A and B) based on differentially expressed gene (DEG) profiles. ICI gene signatures A and B were constructed, comprising genes that exhibited positive or negative associations with the respective clusters. To minimize noise and eliminate redundant genes, dimensionality reduction was applied. Then, we performed the Boruta algorithm to reduce dimension in the ICI gene signatures A and B [22]. For variables of ICI landscape in osteosarcoma patients, PCA was employed to extract the first principal component as the signature score. Construction of ICI score of every patient was built using a method similar to the gene expression grade index [23] with ICI ∑PC1_A_−∑PC1_B_. We classified patients based on ICI scores using the surv‐cutpoint function from the survival package.

### 2.5 Identification and Comparison of Key Genes Based on ICI Score

Weighted gene co‐expression network analysis (WGCNA) was conducted on DEGs and ICI scores using the R package “WGCNA” [24]. First, we utilized the power function to construct the adjacency matrix (AM) of differentially expressed genes (DEGs), selecting an appropriate power index. Subsequently, the adjacency matrix was transformed into a topological overlap matrix. Finally, gene consensus modules were identified and correlated with ICI scores. The mRNAs within the modules exhibiting the highest correlation with ICI scores were selected for prognostic analysis. The prognostic model was built by conducting multivariate COX with the R package “glmnet” [25]. Afterward, ROC curve and AUC were evaluated with the R package “survivalROC” [26].

### 2.6 Statistical Analyses

R software (version 4.2.1) was employed for all statistical analyses. The Wilcoxon test was performed to draw the comparison between two groups, and Kruskal–Wallis test was performed for more than two groups. The Kaplan–Meier plotter was adopted to plot the overall survival curve for the subgroups, and the log‐rank test was performed to evaluate the differences with statistical significance. Spearman analysis was conducted to calculate the correlation coefficient. *p* < 0.05 showed statistical significance.

## 3 Results and Discussion

### 3.1 Identification of ICI Subtypes and Their Clinical Relevance

**FIGURE 2:**
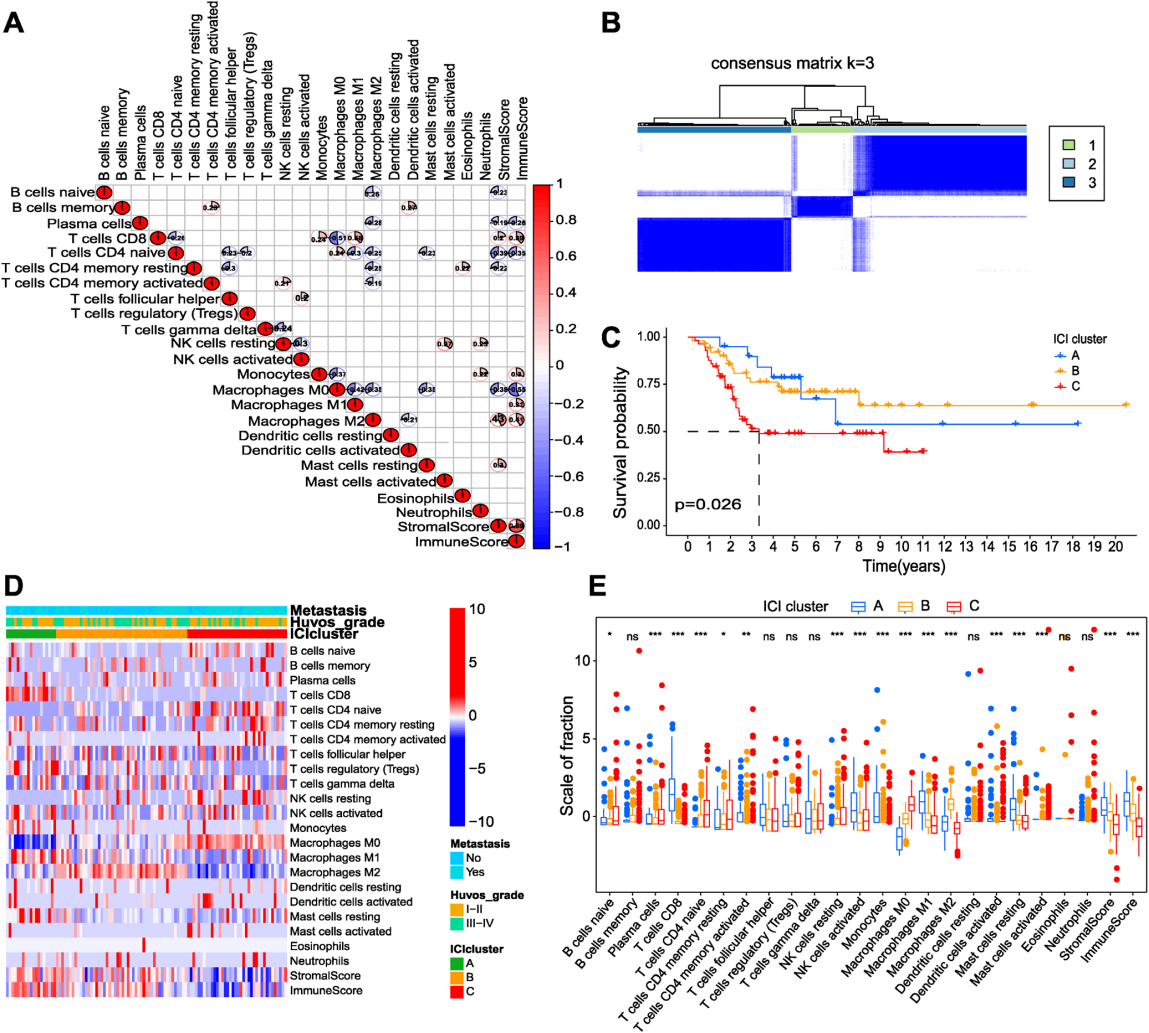
Consensus clustering analysis was conducted on immune cells in osteosarcoma samples, and the characteristics of TME were evaluated. (A) The correlation heat map visualized the universal landscape of immune cell interaction in TME. The correlation coefficient decreased in size from red to blue. (B) Consensus matrixes of all OS samples for appropriate *k* value (*k* = 3), displaying the clustering stability using 1000 iterations of hierarchical clustering. All samples were clustered into three subtypes. (C) Kaplan–Meier curves of overall survival in different ICI clusters. Log rank test showed an overall *p* = 0.026. (D) The heat map depicted unsupervised clustering of ICI in all OS samples. Rows represented tumor‐infiltrating immune cells, and columns represented samples. (E) The fraction of tumor‐infiltrating immune cells, immune score and stromal score in three ICI clusters. The statistical difference of three ICI clusters was compared by the Kruskal–Wallis test. (*p < 0.05, **p < 0.01, ***p < 0.001).

We analyzed 353 osteosarcoma samples (Table S1) to characterize the immune infiltration landscape. Using the “ESTIMATE” and “CIBERSORT” algorithms, we quantified immune cell infiltration (ICI) and visualized interactions via a heatmap (Figure 2A). Results indicated a strong positive correlation between immune score and M1 macrophages, M2 macrophages, CD8+ T cells, and monocytes, while M0 macrophages, plasma cells, and naïve CD4+ T cells showed a strong negative correlation. Based on ICI profiles, consensus clustering identified three distinct subtypes (Figure 2B). Prognostically, Cluster C exhibited significantly poorer prognosis (Figure 2C, *p* = 0.026) and was significantly enriched in metastatic and high‐grade (Huvos III–IV) samples (Figure 2D). Immunologically, this cluster represented an immunosuppressive microenvironment marked by elevated M0 macrophages, naïve B cells, and plasma cells, alongside reduced CD8+ T cells and M1/M2 macrophages (Figure 2E).

### 3.2 Discussion

These findings, illustrated in Figure 2C–E, underscore the clinical relevance of the immune contexture in OS. The poor prognosis associated with Cluster C aligns directly with its immunosuppressive characteristics (shown in Figure 2E), consistent with prior evidence linking M0 macrophages to tumor progression and immune evasion [27, 28, 29]. As precursor cells, M0 macrophages possess the plasticity to differentiate into either phenotype based on microenvironmental factors [30]. Conversely, our observation that overall macrophage abundance is associated with a favorable prognosis is strongly supported by a recent study demonstrating that higher macrophage infiltration levels correlate with improved survival in osteosarcoma patients [31]. This notion—that total macrophage presence is beneficial—is further underscored by the poor prognosis of Cluster C, which exhibited a concurrent reduction of both M1 and M2 populations. This presents a paradigm challenging the conventional M1/M2 dichotomy. While M1 macrophages are traditionally associated with antitumor immunity through pro‐inflammatory cytokine production and antigen presentation [32], and M2 macrophages with pro‐tumor functions including angiogenesis and tissue remodeling [33, 34], their simultaneous decrease suggests that macrophage abundance overall—rather than polarization state alone—may be critical in osteosarcoma progression. This observation implies that specific molecular programs within macrophage populations, particularly the M2 subset, may under certain contexts exert non‐canonical, tumor‐suppressive effects, aligning with emerging concepts of profound functional heterogeneity [35]. This pattern may reflect a process of immune evasion and indicates that certain macrophage densities are necessary for maintaining immune homeostasis. Previous studies have shown that complete macrophage depletion can accelerate tumor progression in some models, likely due to the loss of their tumor‐suppressive and immune‐stimulatory functions, which underscores the beneficial role of a regulated macrophage population [36]. This complex macrophage landscape echoes the “immunoediting” hypothesis where tumors evolve to evade immune recognition [37]. The coordinated loss of multiple immune populations in Cluster C is indicative of advanced immunoediting, resulting in an immune‐barren microenvironment that facilitates metastasis and confers treatment resistance. This ‘immune‐desert’ phenotype, characterized by the paradoxical loss of both pro‐ and anti‐inflammatory macrophages alongside cytotoxic deficits, prompts a deeper investigation into the specific immune regulatory mechanisms at play. For instance, the expansion and potent immunosuppressive function of myeloid‐derived suppressor cells (MDSCs) have been established as a key mechanism of immune evasion in osteosarcoma [38]. Concurrently, the upregulation of immune checkpoints like PD‐L1 on tumor cells can induce T‐cell exhaustion, another key mechanism of immune escape that our gene expression data may reflect [38, 39]. Furthermore, the prominence of M0 macrophages in our poor‐prognosis cluster may implicate the involvement of Myeloid‐Derived Suppressor Cells (MDSCs), a heterogeneous population known to potently inhibit T cell function and promote angiogenesis. Elevated MDSC frequencies in the peripheral blood and tumor microenvironment of OS patients have been consistently linked to metastasis and resistance to conventional chemotherapy [40]. The immunosuppressive patterns we describe likely result from a confluence of these mechanisms: dysfunctional antigen presentation, checkpoint ligand expression, and the recruitment of suppressive myeloid populations like MDSCs, collectively fostering a tolerant microenvironment that facilitates tumor progression and limits the efficacy of immunotherapies. Novel therapeutic platforms, such as copper‐zinc biosensors developed for targeted drug delivery and overcoming chemoresistance, may provide innovative strategies to disrupt this immunosuppressive milieu and enhance treatment efficacy [41, 42]. Overcoming this suppressive milieu may require innovative approaches that precisely target the TME, such as recently developed metal‐based biosensors designed to counteract chemoresistance and modulate immune responses. While these subtypes show clear prognostic utility, their generalizability may be limited by retrospective data. Our paradoxical finding—that loss of both M1 and M2 macrophages defines the most aggressive subtype—highlights the complex and non‐canonical role of macrophages in osteosarcoma and underscores the need to move beyond bulk transcriptomics to decipher functional cellular states. Future studies must employ single‐cell RNA sequencing and spatial transcriptomics to resolve this heterogeneity, defining distinct macrophage subpopulations and their specific interactions within the tumor microenvironment that drive progression and therapy resistance.

### 3.3 ICI‐Derived Gene Clusters and Prognostic Scoring

**FIGURE 3:**
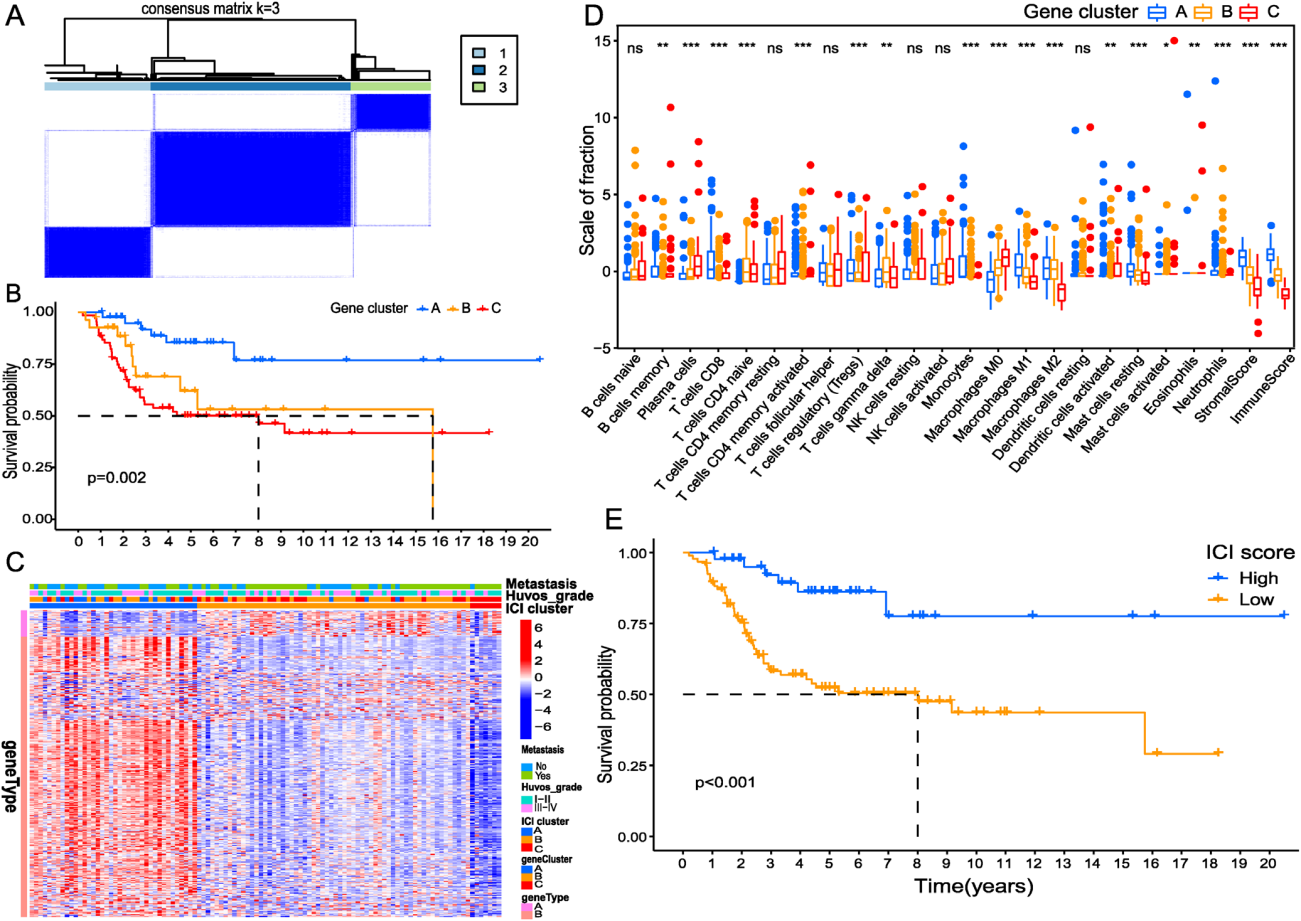
Gene clustering analysis was performed on immune cells in osteosarcoma samples, and a systematic evaluation of the characteristics of TME was conducted. (A) Consensus matrixes of all OS samples for appropriate *k* value (*k* = 3), displaying the clustering stability using 1000 iterations of hierarchical clustering. All samples were clustered into three subtypes based on the DEGs among three ICI clusters. (B) Kaplan–Meier curves of overall survival in different gene clusters. The log rank test showed an overall *p* = 0.002. The heat map depicted the expression of DEGs in different ICI clusters and gene clusters. Heat map colors indicate relative DEGs expression levels. (C) The heatmap depicted the expression of DEGs in different ICI clusters and gene clusters. Heat map colors indicate relative DEGs expression levels. (D) The fraction of tumor‐infiltrating immune cells, immune score and stromal score in three gene clusters. The statistical difference of three gene clusters was compared by the Kruskal–Wallis test (*p < 0.05, **p < 0.01, ***p < 0.001). (E) Kaplan–Meier curves of overall survival in different ICI score. Log rank test showed an overall *p* < 0.001.

We identified 315 differentially expressed genes (DEGs) among the three ICI subtypes using the “limma” package. Unsupervised clustering of these DEGs classified the samples into three gene clusters (A, B, and C) (Figure 3A). Prognostic analysis revealed that patients in gene cluster C had significantly poorer outcomes (Figure 3B, *p* = 0.002). A heatmap illustrated the expression patterns of these DEGs across ICI and gene clusters, showing that gene cluster C was enriched with samples from ICI cluster C, metastatic tumors, and high Huvos grade (III–IV) cases (Figure 3C). This cluster was characterized by high infiltration of plasma cells, regulatory T cells, and M0 macrophages, and low levels of CD8+ T cells, M1 and M2 macrophages, stromal score, and immune score aligning with the poor‐prognosis ICI subtype (Figure 3D). The derived ICI score effectively stratified patients into high‐ and low‐risk groups, with high scores correlating with improved survival (Figure 3E, *p* < 0.001).

### 3.4 Discussion

The strong concordance between gene expression clusters (Figure 3A) and the previously defined ICI subtypes (Figure 2B) reinforces the robustness of an immune‐based classification in OS. This molecular consistency, visualized in Figure 3C, suggests that the transcriptomic landscape faithfully captures the underlying immune biology of the tumor microenvironment [43]. The ICI score (Figure 3E) represents a significant advancement as a continuous quantitative metric of immune activity, providing a more nuanced and clinically applicable tool for prognosis than categorical groupings [44]. Its strong association with survival outcomes underscores its potential for clinical translation in risk stratification and treatment selection. Notably, the immune‐suppressed phenotype of gene cluster C (Figure 3D), characterized by downregulation of antigen presentation pathways and T cell signaling, aligns with the “immune desert” phenotype observed in other solid tumors [45]. This molecular profile, evident in the heatmap (Figure 3C,D), may explain the poor response to immunotherapy in this patient subset and suggests the need for alternative treatment strategies. The development of the ICI score builds upon emerging concepts in cancer immunology that emphasize the importance of quantifying rather than merely qualifying immune responses [46]. Our approach mirrors successful immune scoring systems developed for other malignancies while addressing the unique microenvironmental features of osteosarcoma [47]. The ICI score represents a promising and quantifiable biomarker for risk stratification. However, its clinical translation requires validation in prospective, multi‐institutional cohorts to confirm its robustness and universal applicability. Although the ICI score holds certain potential as a biomarker, it needs to be validated in prospective, multi‐institutional cohort studies to ensure its universal applicability [48]. Future work should be dedicated to standardizing this detection method and integrating it into clinical workflows. Additionally, exploring the correlation between ICI scores and responses to conventional or immunotherapy treatments can provide a mechanistic basis for treatment selection.

### 3.5 Gene Identification and Clinical Associations

**FIGURE 4:**
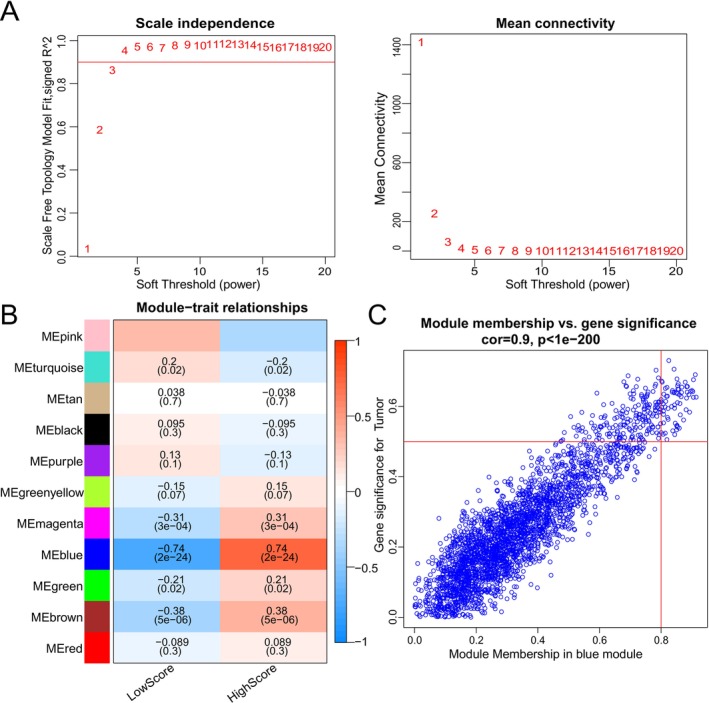
WGCNA analysis between genes and immune cell infiltration signatures. (A) To screen out key genes from the DEGs, a gene co‐expression network was built by using WCGNA to identify important gene modules related to ICI score. By selecting number 3 as the appropriate soft threshold. (B) The blue module contained genes with the strongest correlation and significance to the disease. (C) Module membership (MM) and gene significance (GS) presented significant correlation within the blue module.

**FIGURE 5:**
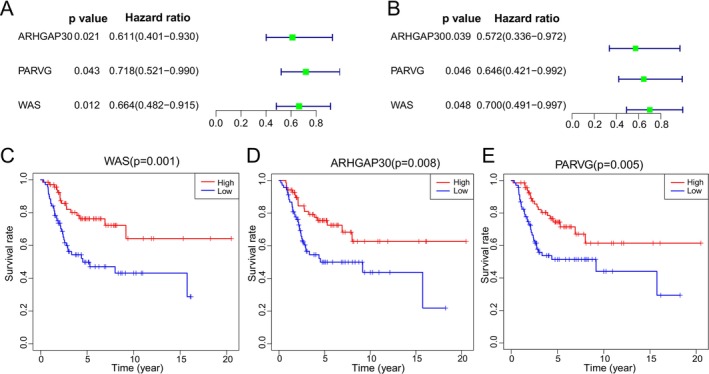
Screening of survival‐related differentially expressed genes. (A, B) The key genes with specific expression patterns based on ICI score, including WAS, ARHGAP30, and PARVG. (C–E) Groups with high expression of WAS, ARHGAP30, and PARVG were significantly associated with improved prognosis (all *p* < 0.05).

**FIGURE 6:**
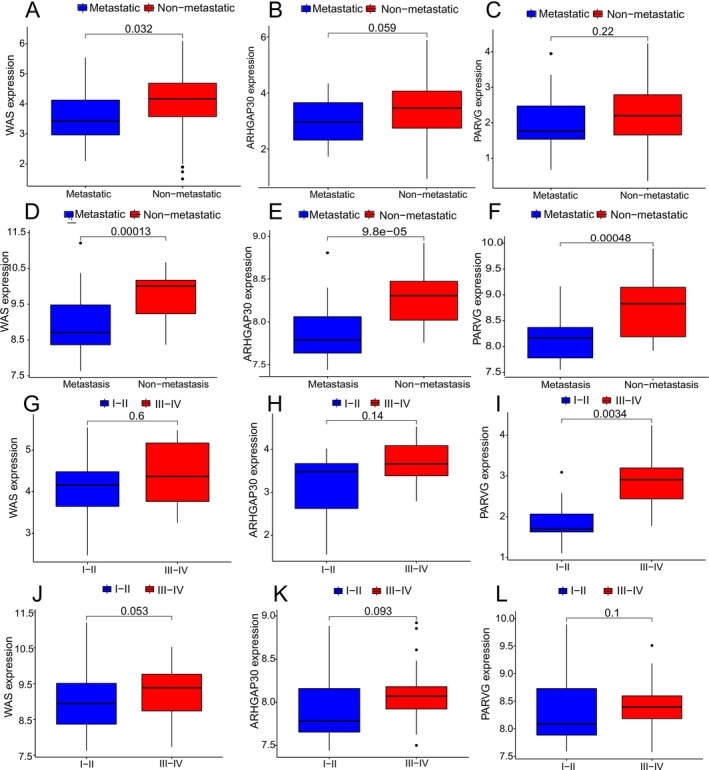
WAS, ARHGAP30, and PARVG were found to be significantly associated with tumor metastasis status and Huvos grade. (A–C) In TARGET dataset, WAS, ARHGAP30 and PARVG were associated with tumor metastasis status. (D–F) In the GSE21257 dataset, WAS, ARHGAP30 and PARVG were associated with tumor metastasis status. (G–I) In TARGET dataset, WAS, ARHGAP30 and PARVG were associated with tumor Huvos grade. (J–L) In the GSE21257 dataset, WAS, ARHGAP30 and PARVG were associated with tumor Huvos grade.

To elucidate key genes underlying the ICI landscape, a weighted gene co‐expression network analysis (WGCNA) was performed. A soft threshold of 3 was selected to construct a scale‐free network, yielding 11 distinct modules (Figure 4A,B). The blue module demonstrated the strongest positive correlation with the ICI score (*r* = 0.74, *p* = 2e−24), with remarkable consistency between module membership and gene significance (*r* = 0.9, *p* < 1e−200; Figure 4C). Through stringent filtering criteria (|GS| > 0.5 and |MM| > 0.8), 70 candidate genes were identified. Subsequent univariate Cox regression analyses in the TARGET and GSE21257 datasets revealed 21 and 6 survival‐associated genes, respectively. Intersection of these results pinpointed three hub genes—WAS, ARHGAP30, and PARVG—whose elevated expression was consistently correlated with improved patient prognosis (all *p* < 0.05; Figure 5A–E). Further clinical correlation analyses demonstrated significantly higher expression of all three genes in non‐metastatic samples across both cohorts (Figure 6A–F). Regarding tumor grade, PARVG exhibited notably higher expression in high‐grade (Huvos III–IV) tumors in the TARGET dataset, with consistent—though statistically non‐significant—trends observed for WAS and ARHGAP30 (Figure 6G–L).

### 3.6 Discussion

The robust identification of WAS, ARHGAP30, and PARVG through integrative bioinformatics approaches reveals crucial insights into osteosarcoma immunobiology. The exceptional correlation between module membership and gene significance (*r* = 0.9) in the blue module (Figure 4C) suggests these genes form a functional complex central to anti‐tumor immunity. Their consistent association with improved survival across datasets (Figure 5C–E) underscores their potential as reliable prognostic biomarkers. The significant downregulation of these genes in metastatic samples across independent cohorts (Figure 6A–F) strongly supports their role as metastasis suppressors. This pattern is particularly significant given the critical need for reliable biomarkers to predict metastatic potential in osteosarcoma. The differential expression patterns relative to tumor grade (Figure 6G–L) reveal important biological nuances, suggesting context‐dependent functionality that may reflect distinct macrophage subpopulations or activation states in different tumor microenvironments [49]. WAS protein regulates actin cytoskeleton dynamics essential for immune synapse formation, phagocytosis, and cellular migration—all critical processes for effective anti‐tumor immunity [50, 51, 52]. The overexpression of WASp has been shown to suppress the growth of non‐small cell lung cancer (NSCLC) [52]. In the context of osteosarcoma, WAS may facilitate immune cell infiltration and activation within the tumor microenvironment. Its association with improved prognosis suggests that enhancing WAS‐mediated immune functions could represent a therapeutic opportunity. In the absence of WASP, both the development and biological functions of M1 and M2 macrophages in the bone marrow are markedly impaired (Biswas et al. 2018). ARHGAP30 is a RhoGAP protein, has been shown to inhibit the Wnt/β‐catenin signaling pathway in lung [53] and pancreatic cancers [54], thereby suppressing tumor progression; it also inhibits the growth of cervical cancer by promoting ubiquitination of NCL and reducing ribosome biogenesis and protein synthesis [55]. The prognostic value of ARHGAP30 expression suggests that dysregulation of Rho signaling pathways may contribute to immune evasion in aggressive OS. PARVG's role in integrin‐mediated signaling suggests its importance in cell‐matrix interactions and immune cell positioning [56]. Some studies suggest that the β and γ isoforms are regarded as putative tumor suppressor genes [57, 58]. The hypomethylation and overexpression of PARVG gene are associated with a better survival prognosis [59, 60, 61]. In osteosarcoma, which originates in the bone matrix, PARVG‐mediated adhesion mechanisms may influence how immune cells navigate and interact with the unique bone microenvironment. The consistent association of PARVG expression with improved outcomes highlights the importance of proper immune cell localization for effective tumor control. The coordinated expression of these three genes across datasets suggests they may function synergistically: WAS regulating cytoskeletal dynamics, ARHGAP30 fine‐tuning GTPase signaling, and PARVG mediating cellular adhesion—together creating an optimal environment for anti‐tumor immunity. This integrated system may represent a vulnerability in aggressive OS, where its disruption could facilitate immune escape and metastasis.

It is important to note that our study did not stratify analysis by patient age, despite known differences in immune responses between pediatric and adult populations [62, 63]. This was due to limitations in the available annotated data. Future studies with well‐annotated, age‐specific cohorts are warranted to validate our findings in different age groups. We argue that while differences exist, the core immune checkpoint pathways we are focusing on the key molecules (such as WAS, PARVG, ARHGAP30) should play a crucial role in the immunotherapy of osteosarcoma, which indicates that the key mechanisms we have studied are to some extent conserved.

While these genes demonstrate strong prognostic value and consistent clinical associations, several important questions remain. The precise mechanisms through which these macrophage‐expressed genes influence metastatic progression require experimental validation using appropriate models. The paradoxical finding of tumor‐suppressive genes enriched in M2 macrophages warrants investigation into functional heterogeneity within macrophage populations. Future studies should employ spatial transcriptomics to map expression patterns within specific tumor niches and investigate the utility of these genes in predicting immunotherapy response. Prospective validation in multi‐institutional cohorts is essential to establish their clinical utility.

### 3.7 The Genes WAS, ARHGAP30, and PARVG Are Predominantly Expressed in Macrophages

**FIGURE 7:**
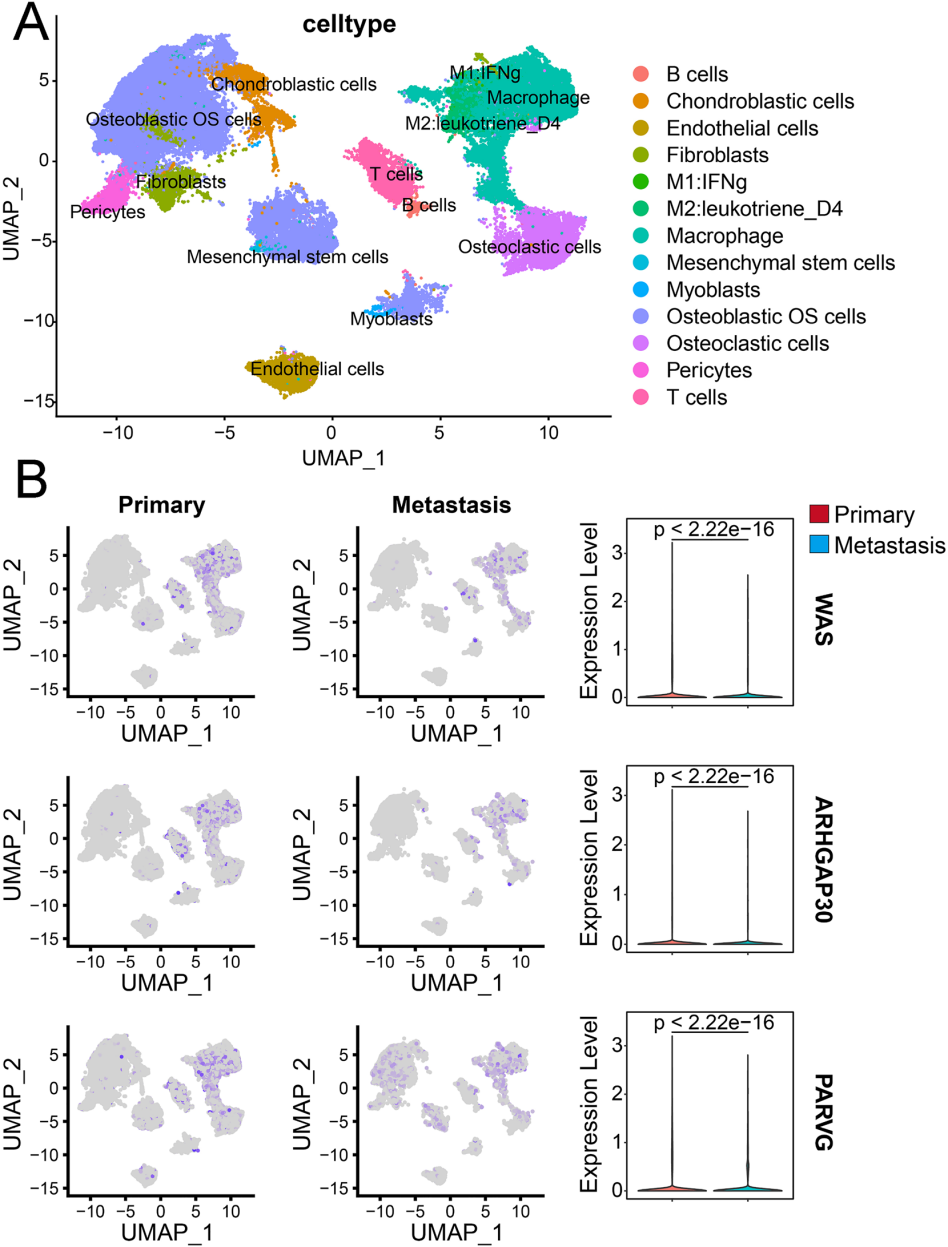
Single‐cell sequencing profiles of primary osteosarcoma and metastatic osteosarcoma. (A) Single‐cell sequencing analysis reveals distinct transcriptional profiles of primary osteosarcoma and metastatic osteosarcoma. (B) The expressions of WAS, ARHGAP30, and PARVG genes are predominantly localized in macrophages, particularly M2‐polarized macrophages. Notably, the expression levels of these three molecules are significantly elevated in primary osteosarcoma samples compared to metastatic tumor samples.

To determine the cellular origin of these hub genes, we performed Single‐cell RNA sequencing analysis of osteosarcoma samples (GSE152048) which revealed that WAS, ARHGAP30, and PARVG were predominantly expressed in macrophages, particularly the M2 subtype (Figure 7A). Notably, the expression levels of these three molecules were significantly elevated in primary tumor patients compared to those with metastatic tumors (Figure 7B), consistent with bulk RNA‐seq results. These findings suggest a potential role for these genes in modulating M2 macrophage polarization. These findings suggest a potential role for these genes in modulating macrophage function.

### 3.8 Discussion

The macrophage‐specific expression pattern of WAS, ARHGAP30, and PARVG, particularly their enrichment in M2 subtypes (Figure 7A), provides crucial cellular context for interpreting their functional roles and the earlier WGCNA and clinical association findings (Figures 4, 5, 6). This localization suggests that the prognostic and metastatic‐suppressive signals associated with these genes originate largely from macrophage populations within the TME. M2 macrophages represent a heterogeneous population with context‐dependent functions, and these genes may define a unique subpopulation with anti‐tumor capabilities [64, 65]. Alternatively, they might regulate macrophage plasticity. The conservation of expression patterns between single‐cell (Figure 7B) and bulk RNA‐seq data (Figure 6A–F) enhances the reliability of these findings and suggests that macrophage‐specific expression drives the overall transcriptional signatures observed in bulk analyses. This connects directly back to the ICI subtypes (Cluster C having low macrophage abundance, Figure 2E) and the poor prognosis associated with low ICI/gene scores (Figure 3B,E) and low expression of these genes (Figure 5). Accumulating evidence demonstrates that WASP is essential for IL‐10‐mediated STAT3 phosphorylation, thereby maintaining the integrity of this signaling cascade and facilitating the proper differentiation and functional homeostasis of tolerogenic macrophages [66, 67]. While the single‐cell data provide important insights into cellular origins, limitations exist. The sample size is limited and requires validation in larger cohorts. The functional consequences of macrophage‐specific expression remain speculative without experimental validation (e.g., macrophage‐specific knockouts). Future studies should employ spatial transcriptomics to map expression within specific TME niches and investigate the utility of these genes as predictive biomarkers for immunotherapy.

## 4 Conclusion

In this study, we comprehensively characterized the immune landscape of osteosarcoma through integrated bioinformatics analyses. Our findings demonstrate: (1) identified three ICI subtypes and developed an ICI scoring system that effectively stratifies OS patients, (2) the development of a robust ICI scoring system that effectively stratifies patients into prognostic groups (3) the discovery of three macrophage‐associated hub genes (WAS, ARHGAP30, and PARVG) as novel prognostic biomarkers associated with improved survival and metastasis suppression. These findings provide insights into OS heterogeneity and may guide immunotherapy strategies.

However, several limitations should be acknowledged. The retrospective nature of our study and reliance on public database analyses limit the generalizability of our findings. The lack of experimental validation for the identified biomarkers and their functional mechanisms represents another constraint. Additionally, the relatively small sample size for certain subgroup analyses may affect the statistical power of some observations.

Future research should prioritize experimental validation of these findings using in vitro and in vivo models to elucidate the functional roles of the identified genes. Prospective, multi‐institutional clinical studies are needed to validate the prognostic value of the ICI score and hub genes. Furthermore, spatial transcriptomics and single‐cell analyses would help resolve cellular heterogeneity and investigate the potential of these biomarkers as therapeutic targets for personalized immunotherapy in osteosarcoma.

## Author Contributions

**Yuerong Wang:** conceptualization, methodology, software, writing – original draft. **Xueni Liu:** methodology, validation, investigation, writing – original draft. **Guojin Xie:** writing, review and editing, visualization, data curation. **Zheqian Li:** conceptualization, methodology, software, data curation, supervision, writing, original draft, writing – review and editing, project administration.

## Funding

The authors have nothing to report.

## Ethics Statement

The authors have nothing to report.

## Consent

The authors have nothing to report.

## Conflicts of Interest

The authors declare no conflicts of interest.

## Supporting information


**Table S1:** The characteristics of clinical samples from osteosarcoma patients.

## Data Availability

The data that support the findings of this study are openly available in figshare at https://figshare.com/, reference number 10.6084/m9.figshare.29539115.
